# The Challenges of Identifying and Classifying Child Sexual Abuse Material

**DOI:** 10.1177/1079063217724768

**Published:** 2017-09-01

**Authors:** Juliane A. Kloess, Jessica Woodhams, Helen Whittle, Tim Grant, Catherine E. Hamilton-Giachritsis

**Affiliations:** 1 University of Birmingham, UK; 2 Aston University, Birmingham, UK; 3 University of Bath, UK

**Keywords:** indecent images of children (IIOC), child sexual abuse material, child pornography, Internet sexual offenses, online child sexual abuse

## Abstract

The aim of the present study was to (a) assess the reliability with which indecent images of children (IIOC) are classified as being of an indecent versus nonindecent nature, and (b) examine in detail the decision-making process engaged in by law enforcement personnel who undertake the difficult task of identifying and classifying IIOC as per the current legislative offense categories. One experienced researcher and four employees from a police force in the United Kingdom coded an extensive amount of IIOC (*n* = 1,212-2,233) to determine if they (a) were deemed to be of an indecent nature, and (b) depicted a child. Interrater reliability analyses revealed both considerable agreement and disagreement across coders, which were followed up with two focus groups involving the four employees. The first entailed a general discussion of the aspects that made such material more or less difficult to identify; the second focused around images where there had been either agreement (*n* = 20) or disagreement (*n* = 36) across coders that the images were of an indecent nature. Using thematic analysis, a number of factors apparent within IIOC were revealed to make the determination of youthfulness and indecency significantly more challenging for coders, with most relating to the developmental stage of the victim and the ambiguity of the context of an image. Findings are discussed in light of their implications for the identification of victims of ongoing sexual exploitation/abuse, the assessment and treatment of individuals in possession of IIOC, as well as the practice of policing and sentencing this type of offending behavior.

In the United Kingdom, Section 160 of the Criminal Justice Act (Home Office, 2003) criminalizes the possession of an indecent photograph or pseudophotograph (i.e., computer-generated) of a child, as well as the taking, making, distributing, and sharing of an indecent photograph or pseudophotograph of a child (Section 1; Protection of Children Act; Home Office, 1999). For these offenses, a “child” is defined as anyone below 18 years of age (UN Convention on the Rights of the Child [UNCRC], 1989). Most European countries now have statutes that criminalize the possession of indecent images of children (IIOC), more commonly referred to as “child pornography” outside of the United Kingdom (Gillespie, 2010; Taylor, Holland, & Quayle, 2001). Furthermore, the UNCRC’s (1989) Optional Protocol on the Sale of Children, Child Prostitution, and Child Pornography requires all parties to prohibit the sale of children, child prostitution, and child pornography; as of June 2016, 173 countries are party to the protocol (OHCHR, 2016). In the United Kingdom, the term “indecent images of children,” rather than “child pornography,” is used to describe indecent photographs or pseudophotographs, as well as moving images (i.e., videos) of children (Sentencing Guidelines Council, 2013).

The sexual exploitation and abuse of children, including the production of IIOC, existed long before the emergence of the Internet; yet, the Internet and other digital technologies have enabled the growing availability of this type of material to users across the world (Quayle & Taylor, 2002), without the personal risks that were previously involved in the physical process of gaining access to such material. These technologies further facilitate the home-based production of IIOC through the use of scanners and digital or web cameras. Coupled with the unique features of the nature of the online environment, namely anonymity, accessibility, and affordability (Cooper, 1998), this highlights the ease with which such material can be accessed, downloaded, distributed, traded, and produced, as well as how Internet technologies in general may be misused by users with ill-intent (e.g., sexually soliciting and grooming children online; Kloess, Beech, & Harkins, 2014; Kloess et al., 2017).

Over recent years, offenses in relation to the production, possession, and distribution of IIOC have assumed great prominence (Babchishin, Hanson, & VanZuylen, 2015; Seto, Hanson, & Babchishin, 2011; Taylor et al., 2001). This has not only led to a number of research studies of individuals who have committed IIOC offenses to better understand the psychological and criminogenic factors underlying this type of offending behavior (e.g., Eke, Seto, & Williams, 2011; Webb, Craissati, & Keen, 2007), but has also been related to an increase in public attention. Policing methods have evolved to tackle this type of crime and include the establishment of child sexual exploitation units that perform reactive and proactive operations against offenders (Taylor et al., 2001), as well as identify and rescue victims of sexual abuse. A precursor to such operations is the identification of IIOC as (a) depicting a child and (b) being of an indecent nature.

## Estimation of Age

Upon the arrest of a person in possession of IIOC, any available electronic devices (e.g., computer desktop, hard drive, mobile phone, and camera) are seized. As part of the subsequent police investigation, these are submitted for digital forensics analysis, which involves designated analysts manually processing the suspect’s digital material to detect any IIOC that have not been identified as “seen/known” by the Child Abuse Image Database (CAID^1^; i.e., CAID facilitates the identification of previously seen and known IIOC based on their unique identifiers, alias hashes). In doing so, the analyst prepares an overview of illegal material found in the suspect’s possession for presentation to the courts in the form of a Streamlined Digital Forensic Report. The report informs the decision-making of the judge regarding the sentence passed on the defendant upon conviction, with images of greater seriousness attracting a longer sentence (Sentencing Advisory Panel, 2002).

In a large number of cases received for digital forensics analysis, the victims in the images will not have been identified (Ratnayake et al., 2014; Sentencing Guidelines Council, 2013). The estimation of the victim’s age therefore lies with the analyst. Furthermore, any “new” image (i.e., an image that is not “known” to CAID) must be recorded in the database, which involves the decision of whether what is depicted within the image meets the legal criteria of IIOC (i.e., the image depicts a child, the image is of an indecent nature). Following this, the image is classified according to the offense categories of A, B, and C.^2^

## Level of Image Severity

With regard to the latter task, several iterations of classification systems have been developed to assist with this process. The COmbating Paedophile Information Networks in Europe (COPINE) scale is a 10-level typology of IIOC (Table 1), ranging from nonerotic and nonsexualized pictures showing children in their underwear or swimming costumes to pictures showing children in a context of sadism or bestiality. This typology was derived from a detailed analysis of more than 80,000 publicly available images that were obtained from newsgroups and websites (Quayle, 2008; Taylor et al., 2001). The scale was originally created as an indicator of how children are victimized through IIOC material; however, it has since been adapted and is used by the courts in England and Wales as a measure of seriousness of the offense, as well as the “dangerousness” of the offender (Quayle, 2008). Initially, the 10-level COPINE scale was adapted to form a classification system of Levels 1 to 5 (Table 1) through the removal of the original COPINE Levels 1 to 3. The reasoning behind this change was that nudity alone (of which varying degrees formed part of the COPINE Levels 1 to 3) was not indicative of indecency. Subsequently, Levels 1 to 5 were further reclassified into a classification system of offense categories (i.e., Category A, B, and C; Table 1), which distinguishes between images involving penetrative sexual activity, images involving nonpenetrative sexual activity, and images of erotic posing (Sentencing Guidelines Council, 2013).

**Table 1.** Overview of the Different Classification Systems.

COPINE Scale (Taylor, Holland, & Quayle, 2001)	
Level 1	Indicative: Nonerotic and nonsexualized pictures showing children in their underwear, swimming costumes, and so on, from either commercial sources or family albums; pictures of children playing in normal settings, in which the context or organization of pictures by the collector indicates inappropriateness
Level 2	Nudist: Pictures of naked or seminaked children in appropriate nudist settings, and from legitimate sources
Level 3	Erotica: Surreptitiously taken photographs of children in play areas or other safe environments showing either underwear or varying degrees of nakedness
Level 4	Posing: Deliberately posed pictures of children fully or partially clothed or naked (where the amount, context, and organization suggests sexual interest)
Level 5	Erotic posing: Deliberately posed pictures of fully or partially clothed or naked children in sexualized or provocative poses
Level 6	Explicit erotic posing: Emphasizing genital areas where the child is posing either naked, partially clothed, or fully clothed
Level 7	Explicit sexual activity: Involves touching, mutual and self-masturbation, oral sex, and intercourse by child, not involving an adult
Level 8	Assault: Pictures of children being subjected to a sexual assault, involving digital touching, involving an adult
Level 9	Gross assault: Grossly obscene pictures of sexual assault, involving penetrative sex, masturbation, or oral sex involving an adult
Level 10	Sadistic/bestiality: (a) Pictures showing a child being tied, bound, beaten, whipped, or otherwise subjected to something that implies pain; (b) Pictures where an animal is involved in some form of sexual behavior with a child
Levels 1-5 (Sentencing Advisory Panel, 2002)	
Level 1	Images depicting erotic posing with no sexual activity
Level 2	Nonpenetrative sexual activity between children, or solo masturbation by a child
Level 3	Nonpenetrative sexual activity between adults and children
Level 4	Penetrative sexual activity involving a child or children or both children and adults
Level 5	Sadism or penetration of, or by, an animal
Offense categories (Sentencing Guidelines Council, 2013)	
Category A	Images involving penetrative sexual activity, possession of images involving sexual activity with an animal or sadism
Category B	Possession of images involving nonpenetrative sexual activity
Category C	Images of erotic posing

Gillespie (2010) argues that while it may be relatively easy to identify clearly indecent images (e.g., photographs of prepubescent children engaged in a sexual act), it becomes more difficult to define images that are less explicit (e.g., photographs of an older adolescent who is partially clothed). In accordance with our point of argument above, Gillespie’s (2010) statement actually represents the two different decisions that law enforcement analysts face: (a) whether an image is of an indecent nature, and (b) whether the image depicts a child. With regard to whether or not an image is of an indecent nature, this can be difficult to determine. While the offense Categories A and B are relatively clear in terms of including images involving both penetrative and nonpenetrative sexual activity, offense Category C (i.e., “erotic posing”) aims to capture other prohibited images that do not fall within A and B. According to the Sentencing Guidelines Council (2013), the term “erotic posing” may be misleading; however, it explains that there are cases where “an image is not posed or ‘erotic’ but could still be deemed indecent, for example, a naked picture of a child not engaged in sexual activity but with a focus on the child’s genitals” (p. 80). Although the identification of a sexual focus within an image may be more straightforward, we would argue that the determination of whether an image is “posed” or “erotic” can be quite challenging, with the legislative definition remaining rather vague here and leaving this aspect open to subjective judgment. Table 1 provides an overview of the three different classification systems.

With regard to the estimation of age, a study by Cattaneo et al. (2009) examined the accuracy with which medical experts (i.e., forensic pathologists, pediatricians, gynecologists) and lay persons were able to determine whether sexually mature females portrayed in pornographic material were in fact a child (i.e., <18 years) or an adult (i.e., >18 years). Both groups performed poorly and medical experts were no better than lay persons at determining age. The results of the study underline the difficulties associated with the assessment of age of individuals at the adolescent, postpubescent developmental stage (i.e., 15-16 years), and those who are sexually mature (i.e., 17 years and older), from digital material. While this is an important finding, the study was limited to including images depicting older adolescents. It is therefore important to extend this work to the full age range of children that can be depicted in IIOC, as well as understand what features affect the ease with which this task is completed.

These difficulties appear to be related to the large interindividual and interpopulation *variability* in age in children’s commencement of sexual development (Mayer et al., 2014; Ratnayake et al., 2014). According to Tanner (1981), “Although all the events of adolescence usually occur together, the age at which they happen varies greatly from one child to another” (p. 49). Given the challenges reported in relation to the estimation of a child’s age, the question arises as to how it can be ensured that IIOC are identified and classified reliably across designated law enforcement analysts as part of such a pivotal process, which can lead to the detection of serious sexual offenses against children. Hebenton, Shaw, and Pease (2009) argue that “the departure of the usage of the COPINE scale from that originally intended may contribute to the crucial point of inter-rater reliability” (p. 433) in the process of analyzing IIOC. It is of paramount importance that two coders/analysts reviewing the same image would classify said image in the same way (Hebenton et al., 2009).

The reliability with which images can be identified as IIOC is not only important for guiding police investigations and informing the sentencing of defendants (Ratnayake et al., 2014), but also for the formulation of assessment and treatment needs of individuals who have been convicted of offenses involving IIOC by practitioners. Furthermore, the identification of IIOC and their classification in terms of level of seriousness can be part of the research process in studies that contribute to our knowledge of offenders who commit contact sexual offenses against children, as well as those who allow and support this type of offending behavior by viewing IIOC (e.g., Glasgow, 2010; Long, Alison, Tejeiro, Hendricks, & Giles, 2016; Taylor et al., 2001).

The study presented here sought to assess the reliability with which images were identified and classified as IIOC, as well as develop a better understanding of the decision-making process law enforcement personnel engage in when undertaking this task through focus group discussions. The aims of the study were therefore twofold: (a) to quantify the level of agreement between law enforcement employees when asked to identify images of an indecent nature and classify them as IIOC accordingly and (b) to conduct focus group discussions to determine where agreement and disagreement in terms of the nature of images occurs, and which aspects or features within them either make the decision-making process easier or more difficult.

## Definitions

In the present article, the developmental stages of “early” and “later childhood” refer to a child’s age of 1 to 6, and 6 to 10 respectively (S. Black, personal communication, 2016, June 30). The term “adolescent” is used in places to specifically refer to the age group of 10- to 16-year-olds; “older adolescent” is used in places to specifically refer to the age group of 14- to 17-year-olds (Mitchell, Finkelhor, & Wolak, 2001). Throughout, the term “victim” refers to any child depicted in the images seized as part of police investigations, leading to the suspect’s conviction. The term “offender” therefore refers to convicted individuals. This term is also used in relation to other adults who are seen to participate in the sexual abuse of a child in the relevant images.

## Method

### Context

The authors of the present article are members of a research team that forms part of a joint collaboration between a U.K. police force and three U.K. universities. This collaboration involves a wider research project that investigates the sexual exploitation and abuse of children via Internet technologies, one aspect of which is the detailed analysis of IIOC. In addition to the research team’s image analyst, four law enforcement employees were recruited as coders due to the sheer volume of data. All four responded to a call from the Technical Intelligence Development Unit (a specialist unit within the police force that deals with cases of online child sexual exploitation), asking for assistance with the analysis of IIOC as part of the ongoing research project, for which they received financial reimbursement per hour worked. The data used in the two studies that form part of the present article stem from one completed case, in which the offender was convicted.

While the wider research project analyzed all digital material available for the cases under investigation, for the purpose of the studies presented here statistical analyses (i.e., Kappa) were performed to assess the interrater agreement between the five coders of their identification and classification of IIOC in the form of image files (i.e., Study 1). This was to ensure that images were coded reliably. These findings led to the development of the second study to examine in detail the complex decision-making process engaged in by law enforcement employees who undertake the difficult task of identifying and classifying IIOC as per the offense categories currently in use (i.e., A, B, and C), and where agreement and disagreement is likely to occur.

### Participants

The five participants who performed the image analysis in Study 1 were female and aged between 29 and 42 years (*M* = 33.40, *SD* = 5.18). One of the participants was employed on the wider research project to undertake the task of analyzing digital material. She had worked for a national police unit dedicated to the investigation of child sexual abuse for more than 8 years, as part of which she also analyzed IIOC. The remaining four participants were law enforcement employees in a confidential unit that provides tactical support to various areas of operation at the police force involved. Their length of service ranged from six to 13 years (*M* = 9.25, *SD* = 3.30). Two of the four participants are detectives, with the other two being civilian staff. While none of the four participants analyzed IIOC as part of their current role or work responsibilities, they deal with sensitive data on a daily basis. One participant was employed as an image analyst in the past. As part of the research project, the five coders coded more than 300,000 images between them.

### Data Collection and Procedure

#### Study 1: Interrater agreement

The five participants performed the identification and classification of IIOC as part of the wider research project, which involved the coding of a differing number of images of the case used here, ranging between 20,000 and more than 300,000 images (*M* = 81,347.60, *SD* = 131,513.34). This comprised the determination of whether or not an image constituted IIOC (i.e., 0 = image not classified as IIOC, 1 = image classified as IIOC). The task was conducted by each participant on an independent basis. Coding took place on a secure site which was the workplace of the law enforcement employees, as well as the research base for the researcher. To emulate the real-world conditions of the task, no time constraints were put on the participants for completing the coding.

Using SPSS, Kappa was calculated to determine the level of agreement for each pair of coders. Due to the variation in number and type of file reviewed by participants, not all images that were identified as IIOC had been classified by all five participants. This was as a result of the image analysis software program displaying the digital material in a variable order for each participant as they began undertaking the task of coding (on the opening of the software program all digital material available for the particular case is shown in a gallery view). The number of dual-coded images per pairwise comparison of participants therefore varied between 1,212 and 2,233.

Prior to the commencement of any coding by the four law enforcement employees, they were introduced to the topic and task by the image analyst. This involved a description of the range of digital material and an explanation of the current classification system of offense categories (i.e., Category A, B, and C), as well as how this system differs from the previous classification system of Levels 1 to 5, which all four law enforcement employees were familiar with. The participants were also informed that they could withdraw and discontinue their involvement in the research project at any time. Frequent monthly sessions were arranged for them with the in-house psychologist and counselor of the Technical Intelligence Development Unit, of which they all made use.

#### Study 2: Focus groups

Following the completion of the interrater agreement analysis, the participants were contacted via email by the first author to enquire as to whether they would be interested in taking part in a study that would involve focus group discussions about their experiences of the challenges and issues around undertaking the task of identifying and classifying IIOC. A participant information sheet was attached to the email with further details about the nature of the study, participant withdrawal, and confidentiality. Two focus group sessions were scheduled between the first author and the four law enforcement employees^3^ within a 4-week period. Prior to the commencement of the focus groups, participants were asked to sign a consent form to confirm their voluntary participation. Participants were aware of the outcome of the interrater agreement analysis prior to participating in the focus groups, as well as that there were some images that were more difficult to identify and others that were less difficult to identify.

The first focus group (1 hr in duration) followed a semistructured interview schedule and involved a more general discussion about whether there are images that are more difficult and less difficult to classify as IIOC, and if this is related to particular aspects or features within an image. Participants were also asked about their personal experiences of what they found challenging about the process, and the types of images with which they experienced indecisiveness.

The second focus group (2 hr in duration) also followed a semistructured interview schedule and involved a detailed discussion about a number of specific images from Study 1. There were 39 images that showed total agreement across coders of an image being IIOC. Of these, 20 images (51%) were selected to be included and form part of the discussion. Furthermore, there were 73 images that showed disagreement across coders in terms of whether or not the image is IIOC. Thirty-six of these images (49%) were also selected to be included. The nature of the images used as part of the discussions varied and ranged from nonindecent to indecent images of the offense categories A, B, and C (i.e., agreement: A [*n* = 3], B [*n* = 9], C [*n* = 8]; disagreement: A [*n* = 3], B [*n* = 4], C [*n* = 22]). While 55 out of the total of 56 images depicted female children, their ages varied and ranged from prepubescent to postpubescent and sexually mature (i.e., agreement: prepubescent [*n* = 17], pubescent [*n* = 3]; disagreement: nonindecent [*n* = 1], prepubescent [*n* = 9], prepubescent/pubescent [*n* = 2], pubescent [*n* = 15], pubescent/postpubescent/sexually mature [*n* = 4]). At least one coder had to have identified the image as IIOC in order for the image to be selected for discussion in the focus group. All images were selected on the basis that they were analyzed by all five coders.

Both focus group sessions were audio-recorded using a Dictaphone and subsequently transcribed by a professional transcription service. The transcribed data were analyzed using thematic analysis. Thematic analysis is “a method for identifying, analyzing and reporting patterns (themes) within data” (Braun & Clarke, 2006, p. 79) across a data set. This method allows for meaningful elements or codes to be combined to generate themes and explanatory models (Guest, MacQueen, & Namey, 2012). The steps undertaken to ensure a rigorous thematic analysis follow recommendations by Braun and Clarke (2006), Guest et al. (2012), and Robson (2011).

Prior to the commencement of coding, the transcripts of the focus group discussions were imported into MAXQDA11, a professional software package with the purpose of facilitating the process of qualitative data analysis. Subsequently, the first author familiarized herself with the data by reading the transcripts in detail. When re-reading the first transcript, any recurrent themes were identified and recorded in the coding scheme by assigning them a descriptive label. These were then organized and ordered into broader themes. The coding scheme was then applied to the remainder of the raw data by highlighting a relevant text and assigning it the appropriate label. Any newly identified themes were added to the coding scheme accordingly.

Where necessary, the broader themes were refined to reflect any additions and ensure that they accurately represented the coded data within them. Finally, overarching themes were developed to arrange thematically similar data together. The coding scheme, its descriptions, as well as the interpretation of the identified overarching themes were developed and revised through discussions with the second and third authors. This was done to ensure that the themes accurately represented the data within them, as well as that they were reliably interpreted.

### Ethics

Full ethical approval for the study was granted by the Science, Technology, Engineering and Mathematics Ethical Review Committee at the University of Birmingham, UK. In addition to this, the research team received vetting clearance to undertake research activities as part of the specialist unit at the UK police force, and adhered to the British Psychological Society (2009) guidelines for ethical practice throughout the contact with the research participants.

## Results

### Study 1: Interrater Agreement

Interrater reliability analyses were conducted on the dual-coded classifications of images as identified by participants. The proportion of agreement was calculated as a percentage of overlap (i.e., κ = kappa). Using the cutoff value of 0.61 (Landis & Koch, 1977), four of the 10 sets of dual-coding reached a level considered to be acceptable. Table 2 presents the κ-values and percentages of agreement for each pairwise comparison.

**Table 2.** Kappa and Percentage Agreement Values for Participants’ Dual-Coding of Images.

	Κ	Number of images	Overall percentage agreement	Percentage occurrence agreement	Percentage nonoccurrence agreement
Pair 1: Coder 1-Coder 2	0.55	1,774	92%	42%	92%
Pair 2: Coder 1-Coder 3	0.73	1,266	95%	60%	95%
Pair 3: Coder 1-Coder 4	0.61	1,698	93%	47%	92%
Pair 4: Coder 1-Coder 5	0.67	2,233	95%	53%	95%
Pair 5: Coder 2-Coder 3	0.55	1,212	93%	41%	93%
Pair 6: Coder 2-Coder 4	0.39	1,815	87%	31%	86%
Pair 7: Coder 2-Coder 5	0.41	1,816	90%	30%	89%
Pair 8: Coder 3-Coder 4	0.56	1,362	93%	42%	93%
Pair 9: Coder 3-Coder 5	0.56	1,241	95%	55%	94%
Pair 10: Coder 4-Coder 5	0.60	1,778	93%	46%	93%

### Study 2: Focus Groups

The qualitative analysis of the focus group discussions revealed a number of factors that appeared to impact on law enforcement employees’ decision-making in the process of identifying IIOC. Participants did not report a particular order in terms of whether they first assessed age and/or indecency within an image, as this depended on the relevant features apparent within each image (e.g., the indecent nature of an image that depicts an adult visibly engaging in sexual activity with a child is immediately obvious versus the indecent nature of an image that depicts a child partially clothed may not be immediately obvious and requires the consideration of additional features to determine indecency).

**Figure 1.**
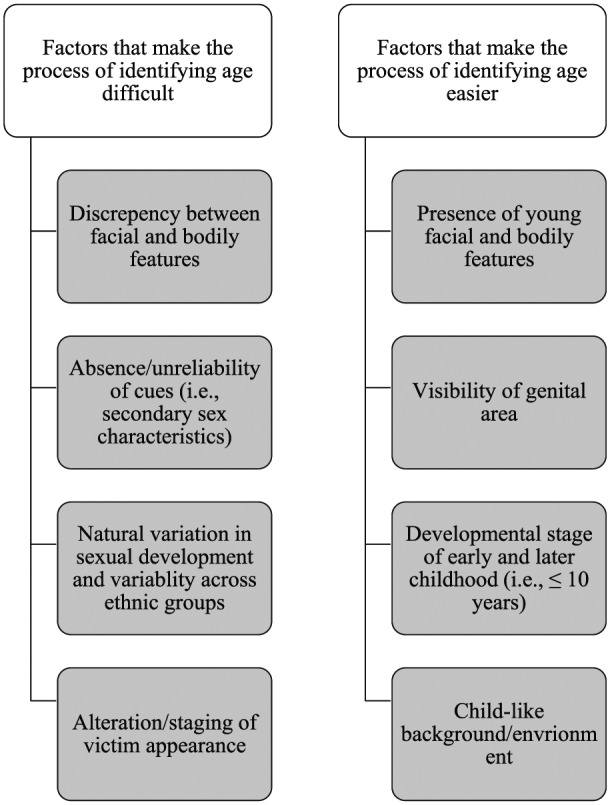
Overview of factors impacting the decision-making process of age.

**Figure 2.**
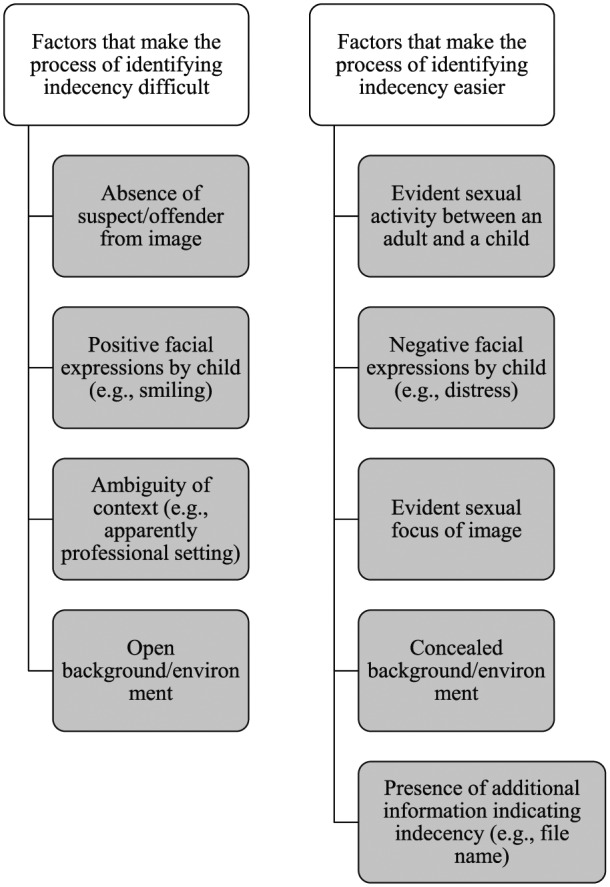
Overview of factors impacting the decision-making process of indecency.

Across the two concepts of age and indecency, a range of five superordinate themes were identified, relating to (a) the appearance of the person depicted in the image; (b) their stage of physical and sexual development; and (c) the composition of the image in terms of portrayal and setting, with the sixth superordinate theme related to the personal conflict participants experienced as part of the decision-making process. The following sections describe the factors that affected the ease with which participants were able to identify whether an image depicted (a) a child and (b) an act of indecency. Figure 1 and Figure 2 provide an overview of these factors.

### Theme 1: Discrepancy Between Bodily and Facial Features

Participants reported experiencing great difficulty with images that depicted children with grown-up facial features, but who did not appear to have reached sexual maturity (i.e., 17 years and older; Tanner, 1981).

I think it’s her hair that makes her look . . . older. (P2)

But I think I probably did look at her face and think that’s an adult. (P1)

Visibly, these images represent a mismatch between a child’s face and body, and participants described the immense difficulty of determining the age of children depicted in such images in light of the natural variation in the development of secondary sex characteristics.

I was starting to struggle around teenage years, because some I thought, oh, they could be 12 or they could be 18. (P1)

She could be a really tall, slim 15-year-old, but she could also be 17. . . . 17, 18, 19, with not very big breasts . . . that’s why it’s difficult, isn’t it, when it’s in that age bracket. (P2)

In contrast, participants’ process of decision-making was facilitated by the presence of both facial and bodily features that indicate youthfulness.

### Theme 2: Presence of Youthfulness

Participants reported that images depicting victims with young features (i.e., facial and body) were easier to identify, both in terms of age and indecency.

I suppose the ones that clearly look like young children, so like babies, toddlers, em, up until probably . . . I was starting to struggle around teenage years . . . So it’s easier to do the ones that are clearly young. (P1)

Specific facial features participants referred to as representing a victim’s young(er) age included larger eyes in comparison with the rest of the face, and dental development (i.e., absence of milk teeth and eruption of adult teeth).

The size of her eyes to the rest of her face, they are still larger than what an adult female’s would be in comparison to her face. (P3)

If you look closely, her teeth aren’t actually fully developed. (P4)

No, she’s got gaps in her teeth. (P2)

While eyes and teeth are perhaps more clear indicators of age, other comments related to features that might be more subjective and representative of lifestyle choices rather than identifying a younger child, such as natural-looking features (i.e., no make-up, undyed hair).

She’s also not wearing any make-up or anything like that . . . Her eyebrows haven’t been done . . . (P1)

Again, her hair is very natural. It’s not been dyed or colored in any way. (P2)

In terms of bodily features, participants described that prepubescent victims at the developmental age of early and later childhood (i.e., ≤10 years) were particularly easy to identify, due to an absence of secondary sex characteristics.

She has no pubic hair. She has no real body shape or breasts that would suggest that she is older . . . (P3)

When you look at her body, she has no breasts, no pubic hair, there’s no feminine shape . . . (P3)

She has got no breasts at all, no development whatsoever. She’s a child. (P4)

Through exposure and/or visibility of a victim’s genital area, participants were able to promptly identify a victim as a child, and an image as indecent in nature respectively. While there are methods that are commonly used for the purpose of pubic hair removal or reduction, the proportions of children’s body parts, as well as their body shape, are still invariably different from sexually mature and physically developed adults (Tanner, 1981). A direct comparison between those is possible in images where the offender is present and depicted in relatively close proximity to the victim.

Especially ones that stood out to me, when you’ve got something else to compare it to, if you get my drift, for scale purposes, you clearly went, yes [that’s indecent]. (P3)

. . . even from the proportion of her legs compared to the man’s legs. (P1)

In comparison to the male, obviously, you can see size-wise that she’s a child. (P4)

You can compare the hands as well, her hand compared to his hand on the back of her head. (P1)

The size of his penis in comparison to her face, it makes her look like a child. (P3)

Because often, as well, you wouldn’t have any other background or anything like that, like it was zoomed in—that’s what you are looking at. Scale and dimension is a good indicator. (P2)

However, particularly during the pubescent (i.e., 11-14 years) and adolescent (i.e., 15-16 years; Tanner, 1981) stages, the determination of a child’s age can prove to be problematic, as individuals vary in the age of onset of puberty and their maturation rates (Stephens & Seto, 2015). As a result of this, some children may appear older in comparison with others of a similar age due to a more advanced physical stage of sexual development. Other children may appear younger due to a less advanced physical stage of sexual development (Cooper, 2011).

The ones that are difficult are when there’s sort of . . . well, it’s the age, isn’t it, whether you are looking at them thinking, well, are you 15 or are you a young-looking 18-year-old, or are you an old-looking 15-year-old, and it’s that area that’s difficult. (P4)

In addition to this, this may further vary across different ethnic groups, especially in relation to body shape and the proportion of certain bodily features (Ratnayake et al., 2014).

Because she’s got quite like a curvaceous . . . like pronounced bottom, which I think would give you the impression sometimes that she’s older. (P3)

### Theme 3: Absence of Reliable Cues

The decision of whether or not a victim depicted in an image was in fact a child could be further exacerbated for participants by the entire absence of the victim’s secondary sex characteristics and/or the unreliability of such cues. A victim’s body parts may not always be visible, particularly in instances, where they do not face the camera.

I think that image is quite hard as well, because you can’t see her genital area. (P1)

So that’s not another determining factor, so you can’t judge that. (P4)

Body parts may also be covered by clothing or other items present in the environment, in which the image was taken. The use of body-enhancing underwear, most commonly in the form of a push-up bra serves a deceptive purpose and prevents the determination of the likely physical stage of sexual development of the victim depicted in the image.

She looks slim build . . . because you’ve got nothing else to go on, have you? Obviously her genital area is covered as well. (P4)

And obviously she is partially-clothed, and she’s got a push-up bra on, but you don’t know whether, because of that, whether she’s underdeveloped or not. (P2)

And her chest could be pushed out rather than being developed to actually exaggerate what’s there. (P2)

In relation to this, the emergence of other, additional items can further alter the appearance of children. Depending on the setting and what the photograph aims to achieve, certain types of clothing are commonly used in the production of IIOC to either reduce or increase the apparent age of the child depicted in the image. Varying pieces of clothing are used for these different purposes, and include the use of make-up, jewelry, lace underwear, and other adult-like clothing to achieve an older appearance, as well as the use of child-like hairstyles and children’s underwear to achieve a young(er) appearance.

And her face looks, like you say, she’s quite made-up. (P3)

She has got that jewelry on, which would make you think she’s more grown-up. (P4)

They have tried to make her look like a child as well. Even though she is a child, they’ve made her look like childish with the tongue out and hair in pigtails. It’s made it more pronounced. (P1)

I think the hair is trying to portray her to look younger than what she is, but facially I don’t think she is that young. (P3)

### Theme 4: Revealing Environment

In instances where the facial and bodily features of a victim depicted in an image do not provide sufficient information, participants are drawn to consider more contextual factors to help them determine whether the victim is a child and if the image is of an indecent nature. Helpful indicators can be derived from the background of the image and the environment in which the image was taken (Cooper, 2011). For example, this can give clues as to the type of room in which the image was taken, and whether it is likely to belong to a child or an adult. Children’s rooms commonly feature child-like bedding and a plethora of toys, as well as colorful and patterned wallpaper.

She’s got posters of [children’s film] in the background. She’s got a hula-hoop and things that an adult wouldn’t have in their bedroom. (P2)

. . . , and playing with cuddly toys. (P2)

The background looks like a bedroom, but not a child’s bedroom.—I’d say that was an adult’s bedroom. (P2)

Other images depict a concealed scene and appear to have been taken in a secluded environment (i.e., an environment that minimizes the likelihood of detection), which in itself may suggest wrong-doing. This includes photographs that are taken covertly, where the victim is unaware that they have been photographed.

That almost looks like it’s in a hotel room . . . (P4)

And it’s in a wooded area that’s quite clearly not a back garden. (P2)

It’s like a voyeuristic photo, isn’t it? (P1)—Yeah, it’s a sneaky picture, she doesn’t know that that’s been taken. (P3)

### Theme 5: Ambiguity of Context

Participants reported that images where the offender is absent and/or not visible were more difficult to classify as being of an indecent nature. This was further exacerbated by an ambiguous context, which includes features that are not obviously indecent in nature and may involve the depiction of naked children in a natural setting. While the image may depict an underage child, the nature of the image is not necessarily indecent if its focus is not sexual (i.e., neither posed or erotic, and no focus on a child’s genitals; Sentencing Guidelines Council, 2013). Natural settings and environments often demonstrate an open background, which implies a lack of secrecy and has the potential for an alternative explanation (e.g., a child on a beach).

But in terms of any sort of sexual nature, actually in the image, there isn’t any. Because it is actually a family photograph. (P1)—Yeah, and in fact, despite the fact that they’re naked, it’s completely unisexual . . . It’s not . . . there’s nothing about it that’s indecent. (P3)

And in terms of the background, is she on a boat? (P2)—A boat, yeah. (P3)

I think, for me, it’s the ones where I can’t work out who’s taken the photograph, like if they are these beach ones. (P3)

This also applies to images where the relationship between the person depicted in the image and the person taking the image is unclear. Photographs that were apparently taken in a professional setting (e.g., a photo studio) were perceived to be equally difficult to determine in terms of indecency, as the context thereof may have been legitimate (e.g., family photo shoot, in the context of a relationship).

It’s difficult when you don’t know the relationship between the person who’s got the photographs and the people in the photo. (P1)

I found some of those quite difficult actually, like the child modeling ones, because there was a few that were like, you know, like pageants, where they dress them to look like adults . . . (P1)

Like some—I don’t like to use the word “professional,” but some of them did look professional, like in a studio or posed. (P1)

While positive expressions (e.g., awareness, confidence, smiling) in photographs can be difficult to interpret in terms of their genuineness (i.e., a genuine expression vs. one that has been “imposed”), one factor that participants used to determine this appeared to be whether or not the photograph contained a sexual focus. Specifically, images that featured substantial nakedness, partial clothing, dressing in adult lingerie, sexual posing, as well as representing a sexualized depiction in general, were more likely to be identified as of an indecent nature than images that did not contain a sexual focus, and were therefore interpreted as innocent.

The underwear as well, it’s not something that a 16-year-old would wear. (P3)

From the pose, you’re drawn straightaway to the genital area . . . (P3)

To me, it’s the way the underwear has been pulled up again. (P2)—And again, it’s got a sexual focus, focusing on the bottom. (P3)

So it’s a sexual purpose, it’s it really? (P4)—Yeah, wearing a fluffy thong. (P2)

Participants noted the ease of being able to classify an image as indecent when it clearly evidenced sexual activity between an adult and a child, and where the victim is showing signs of distress, discomfort, embarrassment, or a vacant expression.

Obviously, the rest of it, when things are going on, unfortunately, they’re very, very easy. You can just whizz through those. (P3)

The majority of those times, for me, it was obvious that the image was indecent, because it was a higher-level image. So there was sexual activity going on, yeah, so that’s why you couldn’t see the child’s face in those ones, wasn’t it, you know? So it was quite a good indicator really. (P4)

It was a lot to do with the action—like if there was something happening in it, I found it easier. They’re clothed, but they’ve been tied up, . . . (P1)— . . . so it’s quite clearly indecent and . . . Or their facial expression, when they’re clearly in some element of distress . . . But they might be fully-clothed. (P2)

At times, participants’ decisions were facilitated by the presence of a file name that was suggestive of the image depicting a young person and/or being of an indecent nature.

I think the photos that have got the name of the website on them as well, they’re a big giveaway. Like “Cute *(female name)*” *that sounds like young.* (P1)

### Theme 6: Inward Conflict

While the previous themes mainly describe aspects and features that were apparent within images, there was also a more personal theme present throughout the focus group discussions. This theme related to the internal conflict participants experienced as part of the decision-making process, and the way they managed images that presented a real challenge to them in terms of deciding whether the person portrayed was in fact a child.

I felt like I was putting more stuff indecent (*i.e., underage*) that time than the time before, and I don’t know why that was. It’s the ones that are a bit borderline I think, and then . . . you kind of . . . I kind of felt like I was doing them a disservice by not putting them as indecent as well, just in case they were. So, probably, on different days, I might have felt a bit differently about how I would categorize them. (P1)

I wrote down a few sort of questionable ones. . . . Yeah, so I would write down the image number and then I’d go back and look at it, but mainly, it was questionable for me because of the age . . . It was the age . . . I wasn’t sure at all, and I would think, right, okay, I’ll go back to it . . . So it was the age, for me. (P4)

## Discussion

The research presented here aimed to assess the reliability with which images were identified and classified as IIOC by law enforcement personnel, followed by focus groups discussions to examine in detail their decision-making process and develop a better understanding of the aspects and features within such material that may make the process more or less difficult. The interrater reliability analysis revealed that there appeared to be certain images that were associated with a higher level of disagreement across coders. These images were then identified and selected, alongside images where coders were in agreement that they constituted IIOC, and presented to coders as part of focus group discussions to find out more about what it was about these images that lead to such disagreement.

Throughout both discussions, the overarching and recurrent theme related to the enormous difficulty coders experienced in determining the age of adolescent (i.e., pubescent) victims depicted in IIOC. Images that depicted young, prepubescent victims in a clearly sexualized context could be easily identified by coders as being of an indecent nature. This was often facilitated by a presence of young facial features, an absence of secondary sex characteristic, and the smaller proportion of body parts (particularly in instances where the offender was present, which enabled a direct comparison). This was much more difficult with images depicting victims either with the earlier physical stages of sexual development, or with the more progressed physical stages involving the development of secondary sex characteristics (Cattaneo et al., 2012; Ratnayake et al., 2014). According to Tanner (1981), the physical sequence of sexual development is much less variable than the age at which they take place, which highlights the natural variation in children during this growth period and the significant challenge it can present for coders in terms of the estimation of age (Mayer et al., 2014; Ratnayake et al., 2014).

The results further reflect statements by law enforcement personnel that they proceed with caution in relation to “borderline images” (i.e., images that do not obviously fall within the definition of IIOC; Wells, Finkelhor, Wolak, & Mitchell, 2007), and “are likely to concentrate on those images which show clearly the guilt of an offender” (Gillespie, 2005, p. 23). This is very much supported by anecdotal remarks from members of the specialist unit at the collaborating police force who undertake the task of analyzing IIOC on a daily basis. One negative implication of this approach, however, is that some victims of ongoing sexual exploitation and abuse will not be identified and rescued.

Another aspect that can impact on coders’ decision-making is the variability in body shape and proportion across victims of different ethnic origins (World Health Organization [WHO], 2006). With the age of onset of puberty decreasing in the developed world (Cooper, 2011), and the impact of ethnic and body mass index variations, this further highlights how the commencement of sexual development can hinder the reliable determination of the developmental stage of a child (Ratnayake et al., 2014). Furthermore, given the international nature of IIOC (i.e., its production taking place across the world), body growth will undoubtedly vary across victims due to a number of heterogeneous factors (e.g., ethnicity, socioeconomic status, nutritional standards; Mayer et al., 2014; WHO, 2006).

A major challenge was further identified in relation to “lower-level images” (i.e., Category C), and their perceived overlap with potentially nonindecent images. In such instances, participants reported the importance of contextual information to facilitate their decision-making. An ambiguous context sometimes led to an internal conflict due to the possibility that some images could have an innocent explanation. The fact that the images were drawn from a case involving a convicted offender may have therefore impacted on participants’ decision-making and affected their interpretation. With depictions of “erotic posing” (i.e., Category C), intent can be difficult to determine, and may hence be more easily interpreted as innocent, particularly in the absence of more reliable contextual factors. It is inherently difficult to identify the offender’s motivation by merely inspecting an image, and there may be circumstances when possessing, taking, making, showing, or distributing an image of a child between 16 and 18 years may be lawful (e.g., in the context of a relationship; Gillespie, 2005, 2010)

Therefore, the question arises as to the level of impact additional knowledge may have on law enforcement personnel’s interpretation and coding of material under review. Does the knowledge about a suspect’s wider collection and possession of a great number of Category A IIOC, beyond the individual image under review, subconsciously affect one’s interpretation of less serious material that is viewed at a later stage? We would argue that participants’ knowledge of additional information, and the consideration thereof as part of the decision-making process, may have contributed to the lower levels of interrater reliability across their coding. However, in turn, the knowledge of file names suggesting a “young nature” may have increased the interrater reliability of the identification of an image as indecent across participants. While it is acknowledged that file names used to label images may be incorrect and therefore misleading, those in the present study did appear to reflect the depiction within the image.

### Practical Implications

#### Policing

The findings revealed in the present study suggest that it would be important to regularly assess the reliability with which IIOC are both identified and classified in practice by law enforcement analysts to shed further light on the aspects and features within images that make this process challenging. Within the CAID, at least two analysts must agree by classifying an image as being of a particular offense category for the image to be confirmed as A, B, or C. However, anecdotal evidence suggests that even among experienced analysts, there can be significant disagreement both in terms of the determination of a child’s age and the level of indecency depicted in a given image.

Additionally, it has become apparent that analysts’ interpretation and decision-making at an earlier stage in the process can be affected by ongoing exposure to a suspect’s collection. The question therefore arises as to the impact both this and additional knowledge of a case may have on analysts’ coding of material under review. Does the knowledge about a suspect’s wider collection and possession of a great number of Category A IIOC, beyond the individual image under review, subconsciously affect one’s interpretation of less serious material that is viewed at a later stage? We would argue that this question merits further empirical investigation.

Also of relevance are the more personal factors that participants perceived to impact on and affect their decision-making. The highly sensitive nature of the area of enquiry undoubtedly brings with it emotional reactions by professionals to the material viewed. The role of emotions in decision-making is well-documented (Loewenstein & Lerner, 2003), and participants discussed their personal level of experience with (e.g., familiarity with the offense categories) and resilience to the material as important factors that could influence their performance, particularly at times of stress. Coders may further draw on (previous) life experience, such as using known children as a reference point in their decision-making, and be guided by empathetic responses toward victims, as well as feelings of apprehension about the potential for disservice to victims, including both misidentifying or failing to identify ongoing sexual abuse.

Overall, these are important factors that need to be taken into consideration when training law enforcement analysts to undertake this difficult task. Specifically, increasing analysts’ awareness of the psychological factors at play will contribute to and develop their psychological mindedness. In addition to this, computer software programs have begun to emerge which market the ability to automatically identify IIOC. While existing programs do not yet appear to be at the required level of sophistication (specialist police unit, personal communication, 2016, June 7), the use of an artificial intelligence computer software program would, in principle, remove both the personal bias and subjectivity human beings implicate in the process unwittingly. Furthermore, by reducing law enforcement personnel’s exposure to such material, this has the potential to protect employees’ mental wellbeing when undertaking an emotionally challenging task over a longer period of time, and thereby significantly alleviate police resources from a cost-benefit perspective.

### Limitations and Future Directions

The sample sizes for both the quantitative analysis (*n* = 5) and the qualitative analysis (*n* = 4) may be considered small; however, the sensitive nature of the area of enquiry limits the availability of law enforcement employees who are able and willing to take part in a study like this. Future research would benefit from utilizing a larger sample and including analysts from digital forensics analysis units in the sampling frame. A comparison of their performance to that of the participants in the present study would shed further light on the contributing role of level of experience with regard to analyzing IIOC (e.g., Do they use and rely on the same cues? How much attention do they pay to contextual information?), and resilience to such material respectively, as well as strategies to help manage particularly challenging material (e.g., Does revisiting an image at a later stage assist with the decision-making process?). In addition to this, having access to information, such as the age of the child depicted in the image, would allow for a more objective interpretation of the interrater agreement results.

## Conclusion

This present study investigated the interrater reliability of coders undertaking the difficult task of analyzing IIOC. The rates of agreement between participants as to which images did or did not constitute IIOC were variable and did not always exceed what is considered to be an acceptable level. Focus group discussions revealed a number of factors that made this process easier or more difficult. It is important to understand the different factors that can impact on this process, and those that are likely to incur varying decisions across analysts, particularly in relation to “borderline” images. Knowledge thereof is not only of relevance for policing and its practices, but also for practitioners and researchers.

## Notes

1. CAID is the United Kingdom’s national child abuse image database that has been developed by the Home Office in collaboration with the police and industry partners to enable the identification and safeguarding of victims, as well as make the investigating thereof faster and more effective.

2. Category A, B, and C represent the offense categories of the current classification system used in the United Kingdom to categorize indecent images of children according to the level of severity depicted within them.

3. The wider research project, of which the inter-rater reliability analyses were part, had been completed when the focus groups were undertaken, at which time the image analyst had secured employment elsewhere and was no longer available.
